# Salzmann Nodular Degeneration in Ocular and Systemic Diseases

**DOI:** 10.3390/jcm13164900

**Published:** 2024-08-20

**Authors:** Anna M. Roszkowska, Claudia Azzaro, Alessandro Calderone, Rosaria Spinella, Domenico Schiano-Lomoriello, Rita Mencucci, Adam Wylęgała

**Affiliations:** 1 Ophthalmology Clinic, Department of Biomedical Sciences, University Hospital of Messina, 98124 Messina, Italy; 2 Ophthalmology Department, Faculty of Medicine and Health Sciences, Andrzej Frycz Modrzewski Krakow University, 30-705 Krakow, Poland; 3 Ophthalmology Unit, Department of Surgery, University Hospital of Messina, 98124 Messina, Italy; s.sara.spinella@gmail.com; 4 IRCCS Bietti Foundation, 00198 Rome, Italy; domenico.schiano@fondazionebietti.it; 5 Eye Clinic, Department of Neurosciences, Psychology, Pharmacology and Child Health, Careggi Hospital, 50134 Florence, Italy; rita.mencucci@unifi.it; 6 Department of Ophthalmology, District Railway Hospital in Katowice, Medical University of Silesia, 40-752 Katowice, Poland; adam.wylegala@gmail.com

**Keywords:** Salzmann nodular degeneration, corneal degenerations, ocular surface inflammation, corneal nodules, corneal flattening, AS-OCT, keratectomy

## Abstract

This review aimed to evidence the predisposing conditions for Salzmann nodular degeneration (SND), where particular attention was paid to its association with ocular and systemic diseases. SND is a rare disease characterized by bluish-white nodules located in the mid-periphery of the cornea, which are otherwise completely clear. SND has been found in association with different systemic and ocular diseases, and it may have unilateral or bilateral presentation. Initial forms are only diagnosed occasionally as they are asymptomatic, whereas, in advanced disease, the visual acuity might be seriously impaired. Although SND is well described, its exact etiopathology is currently still unknown and is frequently misdiagnosed. It is associated with ocular surface inflammatory conditions and previous corneal surgery, and it has been described in different systemic diseases. Diagnosis is clinically based with slit lamp examinations, and instrumental assessments with corneal topography permit one to observe the alterations of the corneal profile, whereas anterior segment–optical coherence tomography (AS-OCT) is used to investigate the stromal depth of the nodules. Therapy might be conservative with the objective of improving the ocular surface homeostasis and surgical outcomes, where the aim is to restore the corneal regularity and visual acuity. Ophthalmologists should pay particular attention when detecting nodules in patients with ocular and non-ocular inflammatory diseases to guarantee the patient a timely diagnosis and a better therapeutic outcome. Additionally, collaboration between specialists who deal with treating patients suffering from disorders potentially associated with SND is recommended.

## 1. Introduction

Salzmann nodular degeneration (SND) of the cornea was described first in 1925 as a rare disease characterized by bluish-white nodules located in the mid-periphery of the cornea [1]. More specifically, it is now considered a progressive, painless degeneration of the subepithelial layers of the cornea, and it is generally caused by previous inflammatory phenomena or trauma of the ocular surface with unknown etiopathogenesis. Through corneal examination, typical findings are represented by raised, cloudy opacities, which are initially whitish and then turn gray-bluish with a total transparency of the surrounding cornea [2,3,4,5,6,7]. The progress to rounded or oblong, and sometimes confluent, nodules is characteristic in the progression of this disease. Though initially asymptomatic, in its advanced forms (with multiple nodules), SND presents clinical symptoms due to epithelial erosions and may result in severe visual impairment [2,3,4,5,6,7].

SND might present as a mono or bilateral disease, mainly affecting adults with a higher prevalence in females, and it has also been found in association with different ocular and systemic diseases [2,3,4,5,6,7]. Although SND is well described, its exact etiopathology is still currently unknown and is frequently misdiagnosed.

In this review, we aimed to provide an update on SND with particular attention paid to its association with the ocular and systemic diseases that might have common underlying factors that play a role in the pathogenesis of these diseases.

## 2. Epidemiology

SND is considered a rare and poorly understood pathology, and it is frequently misdiagnosed; therefore, its actual exact prevalence is not known. SND is found in around 1 case in 2420 patients [1]. The disease can manifest between 4 and 70 years of age, but it specifically presents an onset in two periods, in the fifth and in the eighth decades of life [1,2,3]. In a retrospective study that included 93 patients, Farjo et al. found that, in males, the age of presentation of the disease is approximately at 69 years of age, whereas it presents at around 52 years of age in females [4]. In fact, as has been reported, it is more frequent in Caucasian women aged between 50 and 60 years, affecting both eyes in 50% of cases [4,5,6,7].

## 3. Physiopathology

The SND’s physiopathology remains unknown. What is known, is that nodules start to form by the break at the level of Bowman’s layer and the epithelial basement membrane. Following these alterations, the activated keratocytes migrate anteriorly to the Bowman’s layer, stimulating the migration of stromal fibroblasts that differentiate into myofibroblasts and produce a fibrotic and disorganized extracellular matrix (ECM) [8].

Histopathological studies have evidenced that the epithelium overlying the nodules is made up of transient amplifying epithelial cells (TAC) and cells with a high proliferative activity. These epithelial cells exhibit a high expression of CK19, which is a TAC marker, enolase-alpha and an enzyme indicating high mitotic activity. Instead, the levels of CK 3/12, which are highly expressed in cells that have completed differentiation, and of ABCG2, a typical marker of stem cells, are very low. Therefore, the cells overlying the nodule are neither stem cells nor cells that have already completed differentiation [8]. Matrix metalloproteinases (MMPs), platelet-derived growth factor (PDGF) and transforming growth factor-beta 1 (TGF-β1) are typically expressed in the corneal epithelium in SND. MMP-2, if increased in the corneal epithelium, degrades type 4 collagen, which is the main element of the epithelial basement membrane, and it favors PDGF and TGF-β1 migration into the stroma. These changes allow for the differentiation of quiescent stromal cells in fibroblasts, and the myofibroblasts migrate anteriorly and produce a fibrotic and disorganized ECM that constitutes the nodules [8].

## 4. Etiology and Risk Factors

To date, the etiology and clearly evidenced risk factors of the disease are unknown. Different factors predisposing one to ECM production and deposition in SND have been reported, and they comprise inflammatory conditions of the ocular surface such as dry eye disease, chronic blepharitis related to meibomian gland disfunction, extensive contact lens (CL) wear, phlyctenular keratoconjunctivitis, pterygium, trauma, vernal keratoconjunctivitis, asymmetric distribution of the tear film with partial blinking and/or ocular surface irregularities secondary to inflammation and other degenerative keratopathies [3,5]. Patient often have a history of ocular infections. A higher incidence of SND has been observed in eyes that have undergone corneal refractive surgery or following as consequence to major surgical stresses. In fact, in a study with a cohort of 180 patients, 27% reported a history of previous eye surgery [7,9]. To date, a model of the genetic transmission of the disease has not been universally recognized. Clusters have been identified in family units where several cases have occurred in individuals in different generations. An autosomal-dominant pattern of genetic transmission has been identified in two cases, and this type of inheritance is believed to be attributable to a mutation of a single gene or to a missense mutation of the TFG-β gene [10,11].

## 5. Clinical Signs and Symptoms

**Figure 1:**
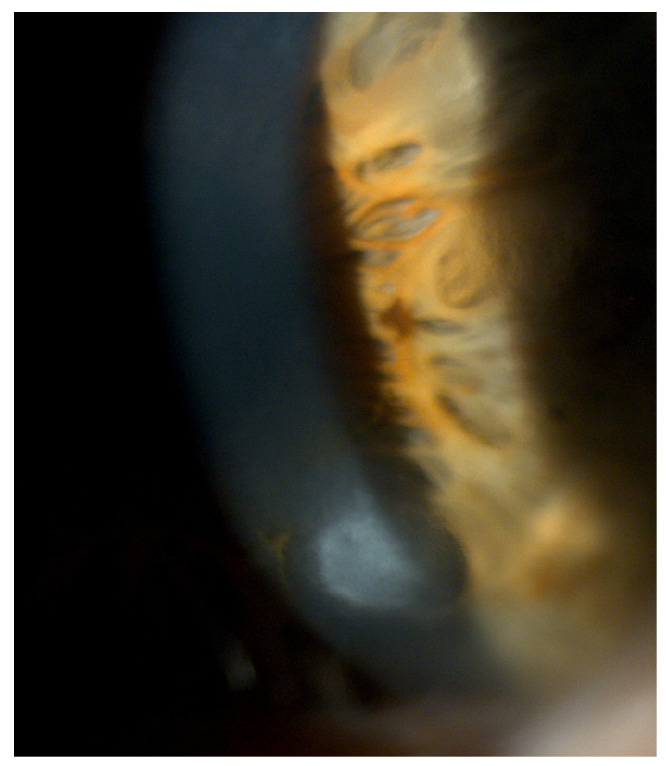
A single nodule.

**Figure 2:**
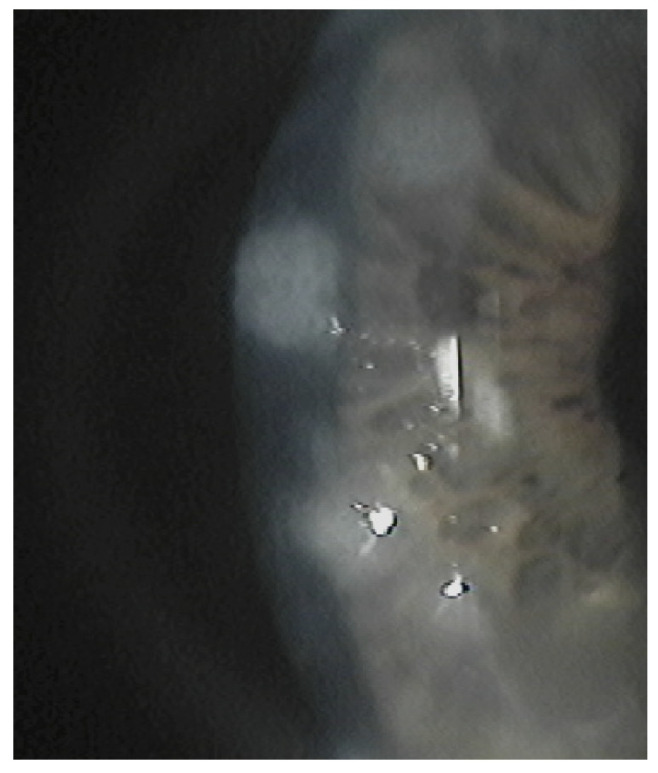
Multiple nodules.

The subepithelial elevated nodules of the disease are gray-white or blue-white, and they often present in a roughly circular configuration involving the central or paracentral cornea. The nodules are typically separated by a clear cornea and are localized in the mid-periphery, and there may be only one or up to eight of them, and the nodules might be confluentin advanced stages.(Figure 1 and Figure 2). In some cases, the nodules are localized in the central cornea. This condition occurs in association with dry eye or long-standing keratitis (e.g., phlyctenulosis, trachoma, and interstitial keratitis), and the degeneration may not appear until years after the active keratitis has subsided. In extensive CL wearers, typical nodular localization at 3 and 9 o’clock has been described [5]. In about 60–68% of patients, there have been reports of symptoms such as pain, foreign body sensation, tearing and relapses in corneal erosions. In advanced stages, the most common presenting symptom is decreased visual acuity, and this is due to induced irregular astigmatism with hyperopic shift.

## 6. Diagnosis

The diagnosis of SND is mainly clinical and is made with a slit lamp examination, where typical nodules of a variable number are observed. The nodules at the slit lamp appear bluish-gray-colored, their shape is rounded and they are sometimes conical or prismatic. The nodules are usually located in the mid-periphery of the cornea, often in the superonasal quadrant. In some cases, the location depends on the associated predisposing factors and on the stress they may induce on the cornea. They are mostly independent from one another but can gather in a circular pattern, resulting in a diffused alteration of the ocular surface.

Furthermore, in these patients, the abnormal distribution of the tear film can be detected by vital dyes.

**Figure 3:**
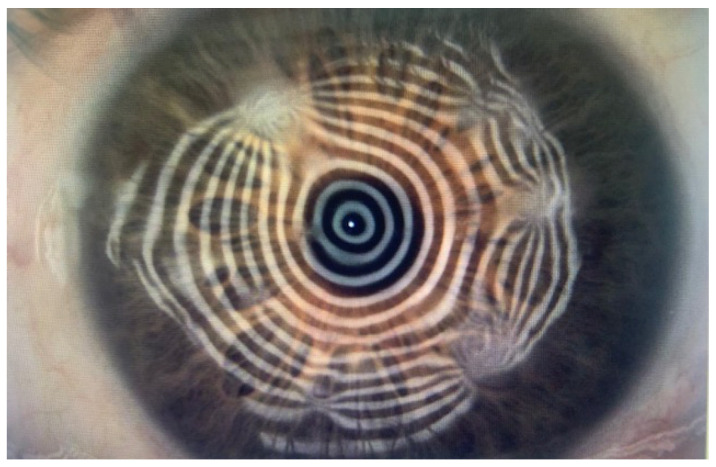
Corneal topography showing surface irregularity of the Placido disk.

**Figure 4:**
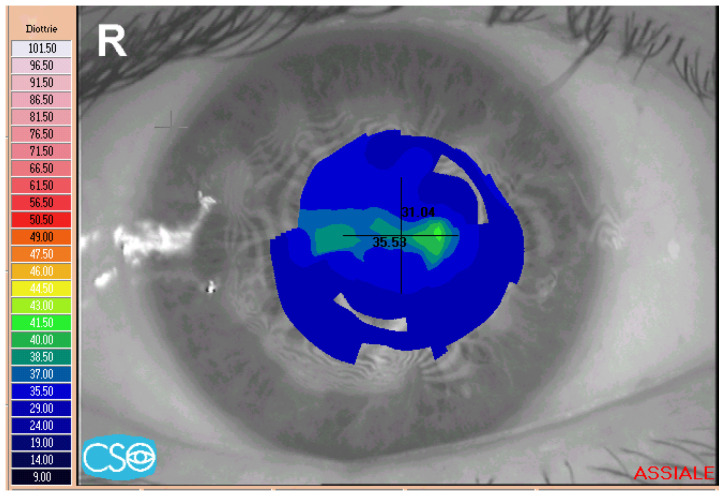
Corneal topography showing an impressive corneal flattening in advanced diseases with multiple confluent nodules.

The corneal topography shows an irregular pattern with steppening in correspondence with the nodules and flattening of the opposite sectors (Figure 3). In advanced SND, when a circular distribution of the nodules occurs, significant central flattening is observed (Figure 4).

AS-OCT can be used to evaluate the morphological characteristics and depth of nodules, as well as their adherence to the underlying planes, in order to take decisive therapeutic choices [12,13]. AS-OCT can show some nodules with triangular spicules that protrude into the underlying space, and they are occasionally shown with a stromal scarring below the Bowman’s membrane.

**Figure 5:**
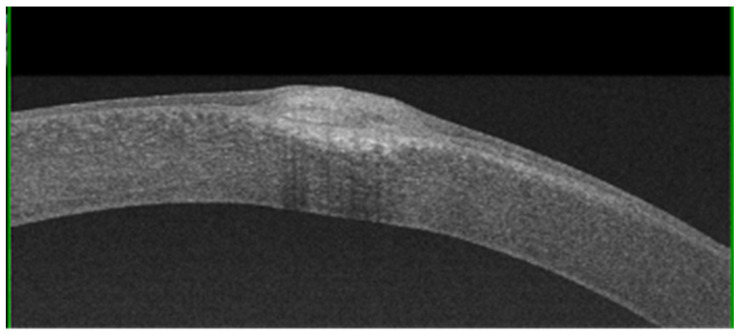
A nodule imaged via AS-OCT showing hyper-reflective deposits located under the epithelium and extending into the anterior stroma.

Additionally, AS-OCT evaluation has proven effective in differential diagnoses with monolateral squamous neoplasia of the ocular surface and in addressing the correct therapeutic approach [14].AS-OCT shows coalescent subepithelial deposits with a variable and heterogeneous signal intensity, probably due to density fluctuations (Figure 5). The nodules are overlaid by a thinned epithelium. A structural and ultrastructural analysis of the corneal epithelium showed dramatic changes; indeed, a reduction in its height, particularly above the nodule in advanced stages, was demonstrated [15].

In vivo confocal microscopy (IVCM) examination cannot evidence specific patterns but it can be useful for providing additional information on the morphological alterations of corneal elements [15]. The epithelial cells in the nodule areas are elongated and polygonal with irregular dimensions. The nodules appear in hyper-reflective and disorganized zones. Sub-basal nerve plexus results are altered with reduced nerve fiber length and density. At the stromal level, the keratocytes appear to be enlarged and there are hyper-reflective nuclei, probably due to the increased cellular activity [16]. The whole stroma appears disorganized, and its deepest part is filled by clusters of keratocytes and an enlarged nerve plexus. The endothelial layer and Descemet membrane result normal.

## 7. Association with Ocular Diseases

Ocular surface disorders together with corneal and anterior segment surgery have been reported as risk factors for the onset of these nodules. Procedures such as penetrating keratoplasty and cataract and refractive surgery have also been reported [17,18,19,20,21,22,23].

As shown in Table 1, Qui et al. reported the first case of SND after a cataract surgery with IOL implantation in both eyes, where a gray-white nodule formed at the clear corneal incision site in a 65-year-old woman 5 years after the procedure [18]. Only a slight sign of the previous corneal incision remained in the right eye, whereas a subepithelial nodule typical for SND was present in the left eye. The authors reported that the diagnosis was made both with AS-OCT and histopathological examination after the superficial keratectomy was performed to reduce the pain of the strong symptoms reported by the patient. The authors hypothesized the appearance of unilateral SND (even though cataract surgery was performed in both eyes) because, in some cases, imperfect corneal incisions might allow for corneal soaking with aqueous humors containing various cytokines, including TGF-b, which activates keratinocytes [24]. If the diagnosis of SND is made before cataract surgery, regardless of the symptoms manifested by the patient, nodulectomy should be performed prior to the surgery, as the presence of the nodule alters the corneal parameters that are required for IOL calculation [25].

Abbott et al. presented the case of a 59-year-old patient affected by superficial Thygeson’s keratitis with symptoms of photophobia, burning and blurred vision [17].

Song et al. reported a case of a 56-year-old woman diagnosed with unilateral SND associated with posterior keratoconus 20 years after penetrating corneal trauma [19].

The appearance of SND after LASIK is thought to be caused by the creation of the corneal flap and has a role in MMP-2, which favors the migration of keratinocytes, which differentiate into fibroblasts and subsequently create deposited in the form of subepithelial nodules [17].

## 8. Association with Systemic Diseases

Little is known about the association of SND with systemic diseases, and, till now, the relationship had not been proved. However, different systemic disease have been reported in patients with SND, and—typically—the diseases have been bilateral in these patients, suggesting a pathogenetic relationship. Table 2 shows the reports on SND in patients with systemic diseases.

Some authors have described the association between dermatopathia pigmentosa reticularis (DPR) and SND [26,27]. DPR is a rare disease with an autosomal dominant pattern of inheritance, and it is characterized by generalized reticulate hyperpigmentation, noncicatricial alopecia and onychodystrophy. One reported case, associated with a moderate dry eye, was bilateral with worsening of visual acuity. The nodules were spread over the entire corneal surface up to the level of the pupillary margin. In this case, the main risk factor was the association between the dermatological condition and the consequential superficial ocular disease.

An association between SND and Crohn’s disease was hypothesized in two case reports [28,29]. Roszkowska et al. described a case of recurrence of previously treated bilateral advanced SND with significant visual loss in parallel with an acute onset of severe Crohn’s disease [28].

Two case reports documented the association of bilateral SND with Kabuki syndrome (KS) [31,32] and, in one case, with Kartagener syndrome [33]. Hopping et al. presented a case of SND in a patient affected by Ehlers–Danlos Syndrome (EDS) [34]. However, the patient had previously undergone LASIK surgery, which, in itself, is already a predisposing factor for SND onset. Yang et al., in their case report, presented a 30-year-old female with bilateral SND and thyroid eye disease [35]. Similar to the previous report, this patient was referred to a bilateral LASIK procedure some years ago. As such, we believe that, in these cases, regardless of underlying systemic diseases, the main risk factor that should be considered is a LASIK surgery aggravated by ocular surface dysfunction.

## 9. Treatment

Therapeutic choices are made regarding the location and the size of the nodules, as well as the severity of the symptoms, e.g., paucisymptomatic cases respond to conservative treatment.

A regime of eyelid hygiene and lubrication of the ocular surface involving warm compresses, massages of the eyelids and artificial tears must be guaranteed. In cases of such therapy being ineffective, patients should be treated with topical steroids, antibiotics and anti-inflammatory or immunomodulatory drugs, such as cyclosporine, or therapeutic contact lenses should be applied. The use of punctual plugs has also been tested in patients with severe dry eye and the presence of severe symptoms attributable to SND.

In cases of significant visual impairment, corneal surgical techniques could be considered with a manual nodulectomy, superficial keratectomy or customized photokeratectomy with an excimer laser (PTK), with or without application of mitomycin-c (MMC) [3,4,36].

A deeper localization of the nodules is correlated with a more difficult excision and to a higher risk of recurrence after nodulectomy; in such cases, a superficial keratectomy is required and is followed by a phototherapeutic keratectomy procedure to smooth the corneal surface with the use of MMC. Lamellar or penetrating keratoplasty procedures are rarely performed as they are only conducted in patients with severe recurrent SND with a deep nodules and loss of VA, and they are frequently associated with other deep corneal alterations [34].

In any case, the therapeutic steps are sequential one to each other if the less invasive ones are ineffective.

The sequential transplantation of amniotic membranes to superficial keratectomy has been evaluated in the prevention of post-operative complications that could favor treatment failure. It guarantees greater safety and speed in healing and visual recovery together with a better outcome in terms of the regularity of the corneal surface, reducing the formation of scars, abnormal neovascularization and inflammatory discomfort [6,37].

## 10. Discussion

In this study, we analyzed the literature on SND, with particular attention being paid to the presence of the disease in local and systemic disorders. The analysis showed that inflammatory ocular diseases induce, in predisposed subjects (probably together with still unknown factors), the formation of nodules in correspondence with the location of the inflammatory foci. In patients affected by systemic pathologies, bilateral involvement has been observed, and, in these forms, recurrences after nodules removal have been reported more frequently due probably to the formation of proinflammatory mediators as result of a generalized alteration of the homeostasis. Only one case, described by Young et al., was affected by Dandy–Walker syndrome, where a group of brain malformations presented with psychotic features, and impulsive and violent behavior were exhibited in a unilateral SND [30]. However, the described nodule was histologically and etiologically different from the other lesions that have been described in the literature. The literature shows a great variability of local and systemic conditions that have been found in patients affected by SND. Although the underlying ocular disorders could be now recognized as a risk factors for SND in predisposed eyes, the impact of systemic diseases is still unknown and should be investigated.

Many authors have studied a possible etiological pathway, albeit without universally agreeing on the risk factors. During this literature review, we gathered the common elements in the lesion development process, combining most common evaluated aspects. Some inflammatory mediators have commonly been traced back to the formation of nodules, but what has not been ascertained is how they can be connected to the predisposing factors. Most authors have identified inflammatory markers as the main element of the vicious circle predisposing the migration of stromal fibroblasts that differentiate into myofibroblasts that produce a fibrotic and disorganized ECM [3].

It is not certain how to prevent the development of lesions, or how the natural history of the disease can be influenced by therapeutic procedures and whether the progression of the nodules can be reversed. The early detection of SND permits starting conservative treatments, where the objective is to improve the ocular surface homeostasis and to potentially reduce the local risk factors and to slow the nodules progression and symptom onset. Additionally, in patients with bilateral SND and no history of ocular diseases, ophthalmologists should search for systemic pathologies. The collaboration between specialists who deal with treating patients suffering from disorders potentially associated with SND is recommended where the aim is to reduce the risk of severe progression and recurrences after nodule removal.

## 11. Database and Literature Search

We used the PubMed, MEDLINE, Scopus, Google Scholar, Web of Science and ScienceDirect databases for the literature search. We searched for “Salzmann”, “Salzmann nodular corneal degeneration”, “Salzmann nodular degeneration”, “cornea”, “superficial keratectomy”, “nodulectomy”, “corneal nodules and systemic diseases”, “ocular disease and corneal nodules”, “ocular surgery and nodules”, “LASIK and Salzmann”, “refractive surgery and Salzmann”.

We included original articles, reviews and case reports that contributed information relevant for this review in the English and German language.

## Figures and Tables

**Table 1:** The association between SND and ocular conditions.

References	Case	Ocular Condition	Nodules Location
Abbott R.L., Forster R. [17]	F, aged 59	Thygeson keratitis	Bilateral, whitish epithelial and subepithelial opacities and grayish subepithelial nodules
Qiu J. et al. [18]	F, aged 65	Previous cataract surgery (Faco + IOL)	Unilateral at the clear corneal incision site
Song P. et al. [19]	F, aged 56	Penetrating corneal trauma	Unilateral
Monaco G., Casalino G. [20]	M, aged 54	MGD treated with intense pulsed light (IPL)	Bilateral in superior quadrants with asymmetric distribution (one large in RE and 5 small nodules in LE)
Moshirfar M., et al. [21] Stem M.S., Hood C.T. [22] He X., et al. [23]	5 Fs, aged between 21–48 M, aged 46	Post-LASIK	Mainly bilateral and located at the peripheric cornea and flap’s margin.

**Table 2:** Association between SND and systemic diseases.

References	Case	Systemic Condition	Location
Goel R., et al. [26] Kammoun S., et al. [27]	F, aged 11	Dermatopathia pigmentosa reticularis	Bilateral spread over the entire corneal surface
Lange A.P., et al. [28] Roszkowska A.M., et al. [29]	F, aged 59 M, aged 43	Chron’s disease	Bilateral middle periphery
Young B., et al. [30]	M, aged 8	Dandy–Walker syndrome	Bilateral middle periphery
Krassin J.G., et al. [31] Martins A. et al. [32]	M, aged 9 M, aged 18	Kabuki syndrome	Bilateral middle periphery
Dugauquier A. et al. [33]	M, aged 46	Kartagener syndrome	Bilateral, symmetrical and flame-shaped in the inferior sector
Hopping G.C., et al. [34]	F, aged 21	Ehlers–Danlos syndrome	Bilateral, and in the nasal and temporal area
Yang M.C., et al. [35]	F, aged 30	Thyroid eye disease	Bilateral
